# Pricing Incentive Mechanisms for Medical Data Sharing in the Internet of Things: A Three-Party Stackelberg Game Approach

**DOI:** 10.3390/s26020488

**Published:** 2026-01-12

**Authors:** Dexin Zhu, Zhiqiang Zhou, Huanjie Zhang, Yang Chen, Yuanbo Li, Jun Zheng

**Affiliations:** 1 School of Cyberspace Science and Technology, Beijing Institute of Technology, Beijing 100081, China; 3220215219@bit.edu.cn; 2 College of Computer Science and Technology, Changchun University, Changchun 130022, China; 231502545@mails.ccu.edu.cn (Z.Z.); 241503541@mails.ccu.edu.cn (H.Z.); 241502527@mails.ccu.edu.cn (Y.C.); 230702309@mails.ccu.edu.cn (Y.L.)

**Keywords:** sensors, medical data sharing, incentive mechanism, social network, game theory

## Abstract

In the context of the rapid growth of the Internet of Things and mobile health services, sensors and smart wearable devices are continuously collecting and uploading dynamic health data. Together with the long-term accumulated electronic medical records and multi-source heterogeneous clinical data from healthcare institutions, these data form the cornerstone of intelligent healthcare. In the context of medical data sharing, previous studies have mainly focused on privacy protection and secure data transmission, while relatively few have addressed the issue of incentive mechanisms. However, relying solely on technical means is insufficient to solve the problem of individuals’ willingness to share their data. To address this challenge, this paper proposes a three-party Stackelberg-game-based incentive mechanism for medical data sharing. The mechanism captures the hierarchical interactions among the intermediator, electronic device users, and data consumers. In this framework, the intermediator acts as the leader, setting the transaction fee; electronic device users serve as the first-level followers, determining the data price; and data consumers function as the second-level followers, deciding on the purchase volume. A social network externality is incorporated into the model to reflect the diffusion effect of data demand, and the optimal strategies and system equilibrium are derived through backward induction. Theoretical analysis and numerical experiments demonstrate that the proposed mechanism effectively enhances users’ willingness to share data and improves the overall system utility, achieving a balanced benefit among the cloud platform, electronic device users, and data consumers. This study not only enriches the game-theoretic modeling approaches to medical data sharing but also provides practical insights for designing incentive mechanisms in IoT-based healthcare systems.

## 1. Introduction

With the rapid development of the Internet of Things (IoT) and mobile health, vast amounts of real-time medical data are continuously collected and uploaded through wearable devices, mobile terminals, and sensors [1,2]. The dynamic health data generated by these daily monitoring devices, when combined with the long-term accumulated electronic medical records and other multi-source heterogeneous clinical data within healthcare institutions, constitute the essential data foundation of intelligent healthcare systems [3]. These rich data resources not only create unprecedented opportunities for personalized diagnosis and disease prediction, but also demonstrate tremendous potential in drug development, public health management, and population-level health interventions.

In a typical Internet of Things data-sharing business model [4], data owners may authorize their data to intermediators for compliant transactions. The intermediator is responsible for data integration, data management, quality auditing, and rule formulation. Data consumers—such as research institutions and biopharmaceutical companies—are allowed to use the purchased data only once within the predefined scope of use, and are prohibited from reselling or further disseminating it. Due to differences in business requirements, various types of data consumers exhibit heterogeneous market demand, leading to different utilities and willingness to pay for the same type of data. Under this structure, a clear causal relationship emerges among data transaction fees, data pricing, and data purchasing volume, which directly determines whether the data-sharing process can form a sustainable incentive loop.

However, existing studies generally exhibit the following limitations. First, most research focuses on privacy protection [5,6], communication security [7], or trustworthy storage technologies [8,9,10], while giving insufficient attention to economic incentives and benefit allocation among multiple stakeholders. Second, many existing models adopt a bilateral structure. For example, some only consider EMR transactions between healthcare institutions and data consumers [11], failing to fully capture patient benefits; others focus on mobile sensing data transactions between data providers and cloud platforms [12], overlooking the central role of data consumers in the value chain; yet others examine EMR transactions between users and research institutions [13], ignoring the platform’s role as a data auditor and its corresponding benefits. These bilateral models do not cover the true three-party interactions present in healthcare scenarios. Third, although some studies introduce social network effects, they lack a systematic modeling of such effects in healthcare data purchasing behavior and do not explain their impact on market equilibrium. Therefore, a systematic theoretical analysis of how intermediators, data providers, and data consumers jointly form a sustainable data-sharing mechanism remains lacking.

Furthermore, in the real-world healthcare data ecosystem, data acquisition, pricing, and utilization exhibit clear sequential and hierarchical characteristics. Intermediators must first establish transaction rules, compliance procedures, and fee structures; data providers then determine their acceptable pricing strategies based on the intermediators’ rules; meanwhile, data consumers’ purchasing decisions may be influenced jointly by the parameters set by the first two parties, budget constraints, data scarcity, and the diffusion effects of social influence. Regulatory requirements and data heterogeneity further reinforce this top-down decision chain, making simultaneous actions, fully competitive settings, or bilateral game structures insufficient to capture the actual business processes. In contrast, the three-party Stackelberg game proposed in this study can accurately model the real-world sequence of “intermediator sets rules → data provider sets price → data consumer makes decision,” and, through backward induction, captures the strategic interactions among hierarchical agents, thus providing a more suitable framework for analyzing such multi-agent incentive mechanisms.

Based on the motivations outlined above, this study proposes a three-party Stackelberg-game-based pricing and incentive mechanism model. This framework naturally captures the hierarchical decision-making chain in IoT healthcare data sharing: the intermediator, as the leader, sets the transaction fee; electronic device users, as first-level followers, determine the data price based on the transaction fee; and heterogeneous data consumers, as second-level followers, decide their purchase volume under the influence of both price and social network externalities. The model not only reflects the power structure and decision sequence present in real-world business operations, but also avoids circular dependencies, allowing the system equilibrium to be derived through backward induction.

The main contributions of this paper are as follows:

(1) A multi-level, three-party Stackelberg game model is constructed to systematically characterize the incentive mechanism in IoT-based medical data sharing. (2) The social network effect is incorporated into the decision-making process of data consumers, revealing its impact on market equilibrium and incentive efficiency. (3) Through theoretical analysis and numerical simulations, it is demonstrates that the proposed model effectively enhances the overall utility of data sharing and achieves a balanced benefit distribution among the intermediator, electronic device users, and data consumers.

This study not only provides a novel theoretical framework for game-theoretic modeling of IoT healthcare data sharing, but also offers practical guidance for designing incentive mechanisms within IoT healthcare systems. The remainder of this paper is organized as follows: Section 2 reviews the related work and current research status; Section 3 presents the mathematical formulation of the three-party Stackelberg game model and defines the corresponding utility functions; Section 4 derives the optimal strategies of all participants and analyzes the equilibrium solutions; Section 5 verifies the effectiveness and parameter sensitivity of the proposed model through simulation experiments; and Section 6 concludes the paper and discusses future research directions.

## 2. Related Work

### 2.1. Incentive Mechanism Based on Stackelberg Game

The Stackelberg-game-based incentive mechanism emphasizes strategic interactions among stakeholders and has been widely applied in scenarios such as mobile crowd sensing, crowdsourcing systems, and data trading. Yu et al. [14] proposed a Three-party Social-aware Incentive Mechanism (TSIM) based on a three-stage Stackelberg game framework, which comprehensively considers users’ social relationships, data quality, and historical reputation to construct the utility functions of requesters, service providers, and mobile users. By applying backward induction, they derived the optimal strategies and proved the uniqueness of the equilibrium. Xiao et al. [15] focused on the design of incentive mechanisms that consider data freshness and social benefits in mobile crowdsourcing systems. They introduced the Age of Information (AoI) metric and constructed an incomplete-information two-stage Stackelberg game model based on AoI guarantees. Furthermore, they proposed an AoI-Aware Incentive mechanism (AIAI) to effectively motivate participants while maintaining data timeliness and social efficiency. Zhu et al. [16] proposed a Fair Data Trading Approach (FDTA), which employs a three-stage Stackelberg game to achieve data value quantification and consignment contract design, ensuring fairness and equilibrium in data trading. However, their work did not account for the influence of social networks among data consumers. Xu et al. [17] addressed the problem of uncertain social sensing workers in spatial crowdsourcing by proposing TACT, an incentive mechanism combining the multi-armed bandit model with a three-stage Stackelberg game, aiming to recruit high-quality workers and maximize the utilities of all participants. Wang et al. [18] proposed a two-stage cooperative incentive mechanism based on the Stackelberg game to solve task allocation and reward distribution problems in mobile crowdsensing, thereby improving both data collection quality and operational efficiency. Zhao et al. [19] designed a two-stage Stackelberg-game-based physical reality sensing incentive mechanism to encourage mobile users to collect physical-world data in the metaverse.

Although the above studies demonstrate the wide applicability of Stackelberg models in mobile sensing and data trading scenarios, their modeling approaches generally rely on two core assumptions: (1) the relationship between leaders and followers is linear or unidirectional, and (2) data demanders do not influence one another. These assumptions make such models more suitable for bilateral structures and limit their ability to capture triadic settings characterized by hierarchical dependence and multi-agent feedback coupling. In addition, when portraying multi-agent complexity, existing studies often simplify the problem by aggregating multiple data consumers into a single overall demand or by assuming independence among the demand-side participants. Such simplifications overlook the widespread social propagation effects observed among real-world medical data consumers, including competitive imitation, academic collaboration, and institutional reputation diffusion. Therefore, although the Stackelberg framework can describe strategic interactions, it still lacks theoretical modeling of how network externalities among multiple data consumers shape equilibrium strategies—a critical gap that the present study aims to address.

### 2.2. Incentive Mechanism Based on Auction Theory

Auction theory [20,21], an important branch of game theory, primarily uses non-cooperative game analysis to study the strategy choices and interaction mechanisms of participants in auction markets, such as auctioneers and bidders, thereby revealing equilibrium outcomes under different rules. Its core objective is to design mechanisms that incentivize bidders to participate based on their true or near-true valuations, ensuring both fairness and efficiency in the auction process. Wang et al. [22] proposed a two-sided auction approach that incorporates user reputation and task preferences to incentivize users to upload high-quality data, thereby improving the availability and accuracy of mobile crowdsensing services. Chen et al. [23] developed a Fairness-Aware Clustering Federated Learning (FACFL) incentive framework, which balances collective and individual fairness. Through a two-level reverse auction design, it optimizes client selection in heterogeneous federated learning scenarios, enhancing model performance. Cui et al. [24] proposed an online auction-based federated learning client selection framework, incentivizing vehicles to participate and thereby increasing the utility of the federated learning platform in vehicular networks. Huang et al. [25] introduced a small-scale, restricted two-sided auction mechanism based on local differential privacy, which uses specific noise-adding strategies and an exponential selection mechanism to protect bidders’ valuation privacy while ensuring both fairness and efficiency in the auction.

Auction mechanisms offer advantages in incentivizing truthful valuation, ensuring efficient resource allocation, and modeling price competition. However, their theoretical foundations largely rely on the assumption that bidders are mutually independent, whereas medical data consumers typically exhibit significant demand interdependence. For example, research institutions’ demand for specific types of medical data is often influenced by collaboration networks, peer competition, and funding trends. The purchasing behavior of one institution may alter the expected benefits of others. Moreover, auction models focus primarily on price discovery and pay comparatively less attention to the strategic role of intermediators in quality control, revenue sharing, and fee adjustment. Therefore, although auctions are suitable for competitive resource-allocation scenarios, they lack the capability to model triadic utility interactions and demand-propagation mechanisms. As a result, auction mechanisms fall short in fully capturing the multi-agent characteristics of medical data transactions.

### 2.3. Reputation-, Contract-, and Blockchain-Driven Incentive Mechanisms

Another line of research emphasizes the design of long-term incentive mechanisms to enhance sustained user participation and engagement. In particular, approaches leveraging reputation systems, contract theory, and blockchain technologies have attracted increasing attention, as they can systematically reward cooperative behavior, ensure fair utility distribution, and provide verifiable enforcement in decentralized or multi-party environments. For instance, Liu et al. [26] proposed an Addictive Incentive Mechanism (AIM) from a behavioral economics perspective to enhance users’ long-term engagement and repeat participation in mobile crowdsourcing tasks. Shen et al. [27] developed a blockchain-based incentive model to promote secure collaborative data sharing across cloud platforms, measuring participants’ marginal contributions using the F1 score and allocating utilities via the Shapley value model. Tang et al. [28] introduced an Effective Interactive Incentive Mechanism with Adaptive Reputation Evaluation (IIM-ARE) to address the unfair reward allocation caused by one-way incentive strategies and non-adaptive reputation models in mobile crowdsensing. Xing et al. [29] proposed a trust propagation–based incentive mechanism to optimize consensus processes in social network group decisions and reduce the total adjustment cost of experts. Chen et al. [30] presented DIM-DS, a dynamic incentive model for federated learning based on smart contracts and evolutionary game theory, which integrates reputation and payment incentives, introduces a cryptocurrency called “reputation coin,” and uses evolutionary game analysis to ensure strategy stability while dynamically adjusting user participation utilities via blockchain smart contracts, promoting more frequent and stable contributions to model training. Gupta et al. [31] designed a contract-theory-based incentive mechanism to motivate intermediate nodes in opportunistic IoT networks to forward information, improving network performance. Fu et al. [32] proposed a federated learning incentive mechanism combined with supervised game theory, enhancing learning accuracy and efficiency in autonomous driving while ensuring data security. Zhang et al. [33] developed a smart contract– and data-quality–driven incentive mechanism to enable IoT devices to safely and efficiently share high-quality data under resource constraints. Liu et al. [34] proposed a blockchain- and trust-based reputation incentive mechanism to increase node participation and system security in smart healthcare systems, effectively mitigating malicious behavior. Wen et al. [35] presented a 6G IoT edge AI content generation incentive mechanism based on diffusion models and prospect theory, innovatively combining contract theory and prospect theory to capture users’ subjective utility and designing optimal contracts with diffusion models, outperforming traditional deep reinforcement learning methods. For multi-stakeholder incentive issues in mobile crowdsensing systems, Yao et al. [36] proposed a utility-based dual-pricing mechanism to balance the utilities of data requesters and participants. Li et al. [37] introduced RATE, a game-theoretic sustainable incentive mechanism for federated learning, which incorporates long-term incentives and leverages a reputation system to optimize utility allocation, effectively promoting continuous high-quality contributions from clients. Building on these approaches, this paper integrates social network effects among data consumers and models the interactions among the cloud platform, electronic device users, and data consumers as a Stackelberg game, while ensuring process continuity, thereby creating an effective IoT-based medical data trading framework.

Reputation-based, blockchain-based, and contract-based incentive mechanisms can effectively regulate long-term behavior, but these approaches typically adopt a perspective of “participant behavior evolution” rather than focusing on the strategic interactions embedded in the market structure itself. Such studies often assume simple data-trading relationships with clearly defined roles and rarely model the strategic dependencies among demand-side participants. In medical data environments, however, data consumers are not only competitors; they are also influenced by collaboration networks, peer-review communities, and the diffusion of scientific communities, all of which create strong social-network correlations in demand. Existing mechanisms still primarily address the management of individual participants’ behavior, lacking hierarchical game-theoretic modeling among three parties and failing to reveal how social influence on the demand side affects equilibrium outcomes. Therefore, while these incentive mechanisms are advantageous for ensuring “long-term trustworthy participation,” they are unable to capture the dynamic strategic interactions among multiple agents that characterize medical data transactions.

### 2.4. Comprehensive Analysis and Theoretical Gaps

Overall, although existing research on incentive mechanisms has developed three major methodological strands—Stackelberg games, auction theory, and reputation- or contract-based approaches—it still exhibits three systematic limitations. First, while Stackelberg games can characterize sequential decision-making and strategic dependence, most models remain constrained to bilateral structures. They thus fail to capture the multi-party transaction chain in medical data markets and overlook how demand coupling among multiple data consumers feeds back into upper-level pricing and contribution decisions. Second, auction mechanisms are effective for modeling price competition and truthful valuation, but they typically assume independence among bidders. In reality, medical data consumers—such as research institutions or hospital alliances—are embedded in collaborative networks where their demands are highly correlated, making the independent valuation assumption implausible. Third, reputation-, contract-, and blockchain-based methods emphasize long-term incentives and credible execution but do not provide a mechanism for strategic propagation among multiple parties, nor can they explain how hierarchical decision-making affects real-time transactions.

More critically, many studies overlook the social network externalities among data consumers. In real medical data markets, research institutions and medical enterprises are often connected through collaboration networks or technical exchange communities. One institution’s purchasing decision may influence the demand of others, leading to diffusion effects and herd-like demand responses. However, existing models rarely incorporate such propagative demand structures explicitly.

Therefore, although the literature has produced important insights into incentive mechanisms, a unified framework that simultaneously captures three-party sequential decision-making and socially influenced demand propagation in medical data sharing remains lacking. To address this gap, the three-stage Stackelberg model proposed in this paper incorporates social externalities among data consumers, thereby filling the above theoretical void and providing a new perspective for analyzing how demand diffusion shapes the decisions of intermediaries and data providers.

## 3. System Model and Problem Formulation

We model the interaction among electronic device users, the intermediator, and data consumers as a hierarchical Stackelberg game, in which each electronic device user determines the unit price of medical data, the intermediator sets the transaction fee, and the data consumers decide their purchasing volumes based on the prices determined by the electronic device users.

### 3.1. System Model

We consider a game system consisting of electronic device users, the intermediator, and a set of socially-aware data consumers. The definitions for each participant are as follows:

**Definition** **1** 
(Data Consumers). *In the medical data sharing system, data consumers are entities that purchase data provided by electronic device users to support purposes such as research, medical analysis, or product development. Data consumers determine the type and volume of data to purchase based on their own needs, budget, and assessment of data value. In this study, we define a set of data consumers C={1,…,j}, where each data consumer j∈C can decide its data purchase volume according to the price set by the electronic device users.*

**Definition** **2** 
(The Intermediator). *The intermediator receives data demand volumes from data consumers and, based on this information, recruits electronic device users to collect their personal measurement data. Meanwhile, the intermediator charges electronic device users a data transaction fee and informs them of the data hosting fee. By reducing the hosting fee, the intermediator can incentivize electronic device users to participate more actively in data sharing.*

**Definition** **3** 
(Electronic Device Users). *Electronic device users refer to individuals who utilize smart wearable devices and other IoT-enabled sensors to monitor and manage their health. Electronic device users provide data for data trading. They determine the unit price of the data and collect the corresponding amount of data as required.*

**Definition** **4** 
(Social Network). *Within the context of a social network, we define the adjacency matrix $G={\left[g_{jk}\right]}_{j,k\in C}$, where each element $g_{jk}$ represents the probability that the data purchase behavior of consumer k affects consumer j. In this model, the social network captures mutual influence at the level of data purchase volumes; that is, a data consumer’s purchasing decision is influenced by the purchase volumes of its social neighbors. The matrix values lie between 0 and 1, i.e., $g_{jk}\in \left[0,1\right]$. If $g_{jk}\neq 0$, the utility of consumer j can increase due to the purchase volume of consumer k, reflecting that neighbors in the social network exhibit similar behaviors due to mutual influence. Consequently, we have $g_{jk}=g_{kj},g_{jj}=g_{kk}=0$, indicating reciprocity in social relationships.*

**Definition** **5** 
(Social Welfare). *We model the social utility as $\sum_{k\in C}g_{jk}t_{j}t_{k}$, representing the additional utility generated by the social network effect. This reflects how increasing a consumer’s data purchase positively influences the purchase level of other data consumers.*

**Definition** **6** 
(Strategy Profile). In the first stage, the intermediator determines its transaction fee strategy based on the market conditions. In the second stage, electronic device users determine their price strategy based on the transaction fees set by the intermediator and the data demand from consumers. Finally, in the third stage, data consumers determine their purchase volume strategy based on their ability to convert data and the prices set by electronic device users. We use $f_{ij}$ to represent the transaction fee strategy for unit data, $p_{ij}$ to represent the price strategy for unit data, and $t_{j}$ to represent the purchase volume strategy of data consumers. The optimal strategy profile $\langle f_{ij}^{*},p_{ij}^{*},t_{j}^{*}\rangle$ ensures that each participant maximizes their utility, resulting in a mutually beneficial outcome. Furthermore, there exists a unique Stackelberg equilibrium (SE), where no participant has the incentive to unilaterally deviate from their optimal strategy.

**Definition** **7** 
(Utility). *We model the utility obtained by different participants to ensure that each party benefits from the data trading process.*

The relevant symbols are described in Table 1.

### 3.2. Problem Formulation

In the context of IoT-based medical data sharing, data transactions typically involve multiple types of participants, including the intermediator, data providers, and data consumers. Their decision-making processes exhibit clear hierarchical structures and characteristics of sequential information disclosure. In existing studies, some works adopt simultaneous-move or non-hierarchical game models to describe multi-party interactions; however, such modeling approaches often fail to capture the actual procedures observed in real-world transactions. Moreover, models that ignore the influence of social relationships among data consumers are generally unable to reflect how collective behavior amplifies or suppresses market demand and incentive effects.

**Figure 1:**
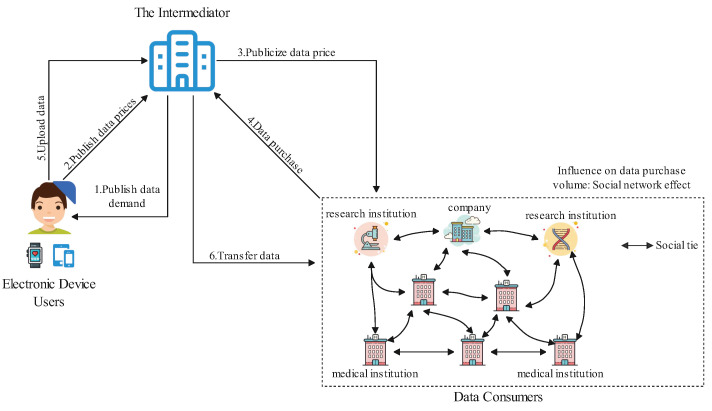
Basic system model for medical data transactions with social network effects.

Therefore, from the perspective of system and mechanism modeling, this paper develops a social-aware incentive framework based on a three-party Stackelberg game, aiming to encourage data consumers to participate in medical data purchases, facilitate the circulation of medical data, and ultimately enhance overall social welfare. In this framework, the intermediator, electronic device users, and data consumers are modeled as decision-makers at different hierarchical levels, allowing transaction rule design, data pricing, and purchasing decisions to be distinguished in both temporal order and information availability. In addition, by explicitly incorporating social relationships among data consumers, the model captures the interdependence of individual decisions within a social network, thereby providing a more accurate characterization of market equilibrium under multi-party interactions. The corresponding transaction process is illustrated in Figure 1. The specific process is as follows:

Step 1: As the leader, the intermediator announces the data demand, the transaction fee, and the data hosting fee to electronic device users (e.g., users of wearable devices or mobile terminals). The intermediator also discloses the categories of data needed and information regarding the intended uses of potential data consumers.

Step 2: The electronic device user sets and submits the unit data price to the intermediator. In the proposed trading mechanism, the device user is informed of the data demand categories and the intended usage of potential data consumers announced by the intermediator prior to pricing, enabling a more informed assessment of the data’s value.

Step 3: The intermediator publishes the data price to potential data consumers, enabling them to make informed purchasing decisions.

Step 4: The After the prices are announced, each data consumer determines its data purchasing volume based on its own demand as well as the purchasing decisions of other consumers (i.e., social network influences), and then reports this decision to the intermediator.

Step 5: Once the intermediator receives the purchase request, it coordinates the data interaction and transmission process. The intermediator sends a data upload request to the electronic device users.

Step 6: After the electronic device users complete the data upload, the intermediator securely and efficiently transmits the data to the respective data consumers.

It is important to note that data consumers are not isolated; rather, they are part of a complex social network. This social relationship network not only affects the purchasing willingness and decisions of data consumers but also has the potential to create chain reactions, impacting the equilibrium of the entire market. Therefore, the introduction of social relationships makes the transaction mechanism more aligned with the group behavior characteristics in real-world scenarios, better reflecting the dynamic game process under multi-party interactions.

Based on the detailed steps outlined in the system’s data transaction model, we formulate the optimal strategy profile as follows. The formation of the optimal strategy profile is derived from a three-stage game framework involving sequential decisions by the three entities.

Stage I (The intermediator): In the first stage, the intermediator, as the leader, determines the optimal transaction fee $f_{ij}^{*}$ after receiving the specific data purchase volume from the data consumers.

Stage II (Electronic Device Users): In the second stage, electronic device users, as the second leader, calculate the optimal unit data price strategy $p_{ij}^{*}$.

Stage III (Data Consumers): In the third stage, data consumers, as followers, determine their optimal purchase volume strategy $t_{j}^{*}$ based on the unit data price $p_{ij}^{*}$ set by the electronic device users.

The objective is to determine the optimal strategy profile $\langle f_{ij}^{*},p_{ij}^{*},t_{j}^{*}\rangle$, ensuring that no participant has an incentive to unilaterally deviate from their optimal strategy to improve their utility, i.e.,

(1) $$U_{pl}\left(t_{j}^{*},f_{ij}^{*}\right)\geq U_{pl}\left(t_{j}^{*},f_{ij}\right)\;(\mathrm{Stage}\;I:\;\mathrm{The}\;\mathrm{intermediator})$$

(2) $$U_{i}\left(t_{j}^{*},p_{ij}^{*},f_{ij}^{*}\right)\geq U_{i}\left(t_{j}^{*},p_{ij},f_{ij}^{*}\right)\;(\mathrm{Stage}\;\mathrm{II}:\;\mathrm{Electronic}\;\mathrm{Device}\;\mathrm{Users})$$

(3) $$U_{j}\left(t_{j}^{*},p_{ij}^{*}\right)\geq U_{j}\left(t_{j},p_{ij}^{*}\right)\;(\mathrm{Stage}\;\mathrm{III}:\;\mathrm{Data}\;\mathrm{Consumers})$$

These inequalities indicate the equilibrium of the Stackelberg game, which is defined as follows:

**Definition** **8** 
(Stackelberg Equilibrium). The optimal strategy profile $\langle f_{ij}^{*},p_{ij}^{*},t_{j}^{*}\rangle$ forms a Stackelberg equilibrium of the game when the inequalities hold.

Our study adopts a three-stage Stackelberg game. By integrating game theory into the IoT-based medical data sharing system, we aim to simultaneously maximize the utilities of the intermediator, electronic device users, and all data consumers.

## 4. Mechanism Design

### 4.1. Utility Function Design

We model the IoT-based medical data sharing and trading problem as a three-stage Stackelberg game to determine the optimal strategy set $\langle f_{ij}^{*},p_{ij}^{*},t_{j}^{*}\rangle$. The intermediator influences the revenue of electronic device users by adjusting the transaction fee $f_{ij}$. Electronic device users adjust the unit data price $p_{ij}$ to affect the utility of data consumers, while data consumers influence the utility of electronic device users by deciding their data purchase volume $t_{j}$. The utility functions for each party are designed as follows:

Utility of Data Consumers: The utility of each data consumer is the sum of data utility and social utility, minus the total cost of purchasing data. The utility function of data consumer *j* is given by

(4) $$U_{j}=\delta \left(\alpha_{j}t_{j}-\beta_{j}t_{j}^{2}\right)-p_{ij}t_{j}+\sum_{k=1}^{|C_{-j}|}g_{j,k}t_{k}t_{j},$$

where δ is an adjustable parameter representing the monetary equivalent value of the consumer’s data purchase, and $\alpha_{j}>0$, $\beta_{j}>0$ are coefficients capturing the concavity of the function. Similar to [12], we adopt a linear-quadratic function to represent tractability, converting the data purchase volume of a data consumer into its monetary utility. This function captures the characteristic of diminishing marginal utility: while the total utility increases as the total purchased data increases, the marginal utility decreases. The term $\sum_{k=1}^{|C_{-j}|}g_{j,k}t_{k}t_{j}$ represents additional social benefits obtained through information sharing among socially connected consumers *j* and *k*. The term $p_{ij}t_{j}$ is the total cost of purchasing data. This linear-quadratic form reflects diminishing marginal utility: while the total utility increases with purchased data, the marginal utility decreases.

Utility of Electronic Device Users: The utility of an electronic device user is the sum of payments from data consumers and social benefits, minus the total cost including transaction fees paid to the intermediator, storage costs, and data collection costs. The utility function of electronic device user *i* is

(5) $$U_{i}=\sum_{j=1}^{\left|C\right|}\left(p_{ij}-f_{ij}\right)t_{j}+\sum_{j=1}^{\left|C\right|}\left(\lambda t_{j}-\mu t_{j}^{2}\right)-\sum_{j=1}^{\left|C\right|}\left(c+b\right)t_{j},$$

where $f_{ij}t_{j}$ denotes the total transaction fee paid by the electronic device user to the intermediator. The term $\left(\lambda t_{j}-\mu t_{j}^{2}\right)$ represents the social benefit gained from sharing data, modeled as a linear-quadratic function. The coefficients λ and μ capture the marginal effect of social benefits. $ct_{j}$ denotes the comprehensive cost of data collection, and *c* is the comprehensive cost per unit of collected data. $bt_{j}$ denotes the data hosting fee paid by electronic device users to the intermediator, and *b* is the hosting fee per unit of data.

Utility of the intermediator: The utility of the intermediator is the sum of transaction fees and hosting fees received from electronic device users, minus the data storage and transmission costs. The intermediator’s utility function is

(6) $$U_{pl}=\sum_{j=1}^{\left|C\right|}\left(f_{ij}t_{j}-st_{j}\right)+\sum_{j=1}^{\left|C\right|}\left(b-w\right)t_{j},$$

where $st_{j}$ denotes the transmission cost, with *s* being the unit transmission cost, and $wt_{j}$ represents the storage cost, with *w* being the unit data storage cost.

### 4.2. Determination of the Optimal Strategy Set

We employ the backward induction method to analyze the three-stage game and obtain the optimal strategy set. In the third stage, each data consumer determines the optimal purchase volume $t_{j}$ based on the given unit data price $p_{ij}$. In the second stage, we determine the optimal unit data price strategy $p_{ij}$ that maximizes the utility of electronic device users. Finally, in the first stage, considering the total data purchase volume of all data consumers, the intermediator determines the optimal transaction fee $f_{ij}$. Furthermore, we prove that the strategy set $\langle f_{ij}^{*},p_{ij}^{*},t_{j}^{*}\rangle$ constitutes a unique Stackelberg Equilibrium (SE), ensuring that no participant has an incentive to unilaterally deviate from their optimal strategy.

**Theorem** **1.** 
*In Stage III, given any unit data price $p_{ij}$, the optimal strategy (i.e., optimal purchase volume) for each data consumer can be determined as follows:*

(7) $$t_{j}^{*}=\frac{\delta \alpha_{j}-p_{ij}}{2\delta \beta_{j}}+\frac{\sum_{k=1}^{|C_{-j}|}g_{j,k}t_{k}}{2\delta \beta_{j}}$$

**Proof.**  We calculate the first- and second-order derivatives of $U_{j}$ with respect to $t_{j}$ as follows:

(8) $$\frac{\partial U_{j}}{\partial t_{j}}=\delta \left(\alpha_{j}-2\beta_{j}t_{j}\right)-p_{ij}+\sum_{k=1}^{|C_{-j}|}g_{j,k}t_{k}$$

(9) $$\frac{\partial^{2}U_{j}}{\partial t_{j}^{2}}=-2\delta \beta_{j}<0$$

Based on (8) and (9), the utility function of each data consumer is strictly concave in the feasible domain of $t_{j}$. The optimal strategy is obtained by solving $\frac{\partial U_{j}}{\partial t_{j}}=0$, and the purchase volume $t_{j}$ is derived accordingly.   □

As shown in Theorem 1, the purchase volume of each data consumer consists of two parts. The first term, $\frac{\delta \alpha_{j}-p_{ij}}{2\delta \beta_{j}}$, is associated with the data utility coefficients $\alpha_{j}$ and $\beta_{j}$, as well as the adjustable parameter δ. The second term, $\frac{\sum_{k=1}^{|C_{-j}|}g_{j,k}t_{k}}{2\delta \beta_{j}}$, captures the potential social network effect that depends on other data consumers.

Although the purchasing strategy of each data consumer is described by (7), if the purchase level of other consumers is sufficiently high, an individual data consumer may increase its own purchase volume without bound. Therefore, the existence or uniqueness of a Nash equilibrium cannot be guaranteed. To address this issue, we propose a sufficient assumption that ensures the existence and uniqueness of the Nash equilibrium described in Theorem 1. Given the budget constraints and limited data processing capability of each consumer, this assumption is reasonable.

**Assumption** **1.** 
$\sum_{k=1}^{|C_{-j}|}\frac{g_{j,k}}{2\delta \beta_{j}}<1,\;\forall j.$

**Theorem** **2.** 
*Under Assumption 1, we can ensure the existence and uniqueness of the Nash equilibrium in Stage III of the Stackelberg game.*

**Proof.**  We first prove existence. Let $t_{j}^{*}$ denote the maximum purchase volume among all data consumers, i.e., $t_{j}^{*}\geq t_{k}^{*}$. Then,

(10) $$\begin{array}{cc} t_{j}^{*} & =\frac{\delta \alpha_{j}-p_{ij}}{2\delta \beta_{j}}+\frac{\sum_{k=1}^{|C_{-j}|}g_{j,k}t_{k}}{2\delta \beta_{j}}\\ \; & \leq \frac{\delta \alpha_{j}-p_{ij}}{2\delta \beta_{j}}+\frac{\sum_{k=1}^{|C_{-j}|}g_{j,k}t_{j}^{*}}{2\delta \beta_{j}}\\ \; & \leq \frac{|\delta \alpha_{j}-p_{ij}|}{2\delta \beta_{j}}+t_{j}^{*}\frac{\sum_{k=1}^{|C_{-j}|}\left|g_{j,k}\right|}{2\delta \beta_{j}} \end{array}$$

Based on (10) and Assumption 1, since $t_{j}^{*}$ is the maximum purchase volume, we can derive $t_{j}^{*}\leq \frac{|\delta \alpha_{j}-p_{ij}|}{2\delta \beta_{j}\left(1-\frac{\sum_{k=1}^{|C_{-j}|}\left|g_{j,k}\right|}{2\delta \beta_{j}}\right)}.$ By considering the data consumers’ optimal purchase-volume strategy, the optimal purchase volume at the upper bound can be determined as:

(11) $$t_{j}^{*}=\max\left\{0,\frac{\delta \alpha_{j}-p_{ij}}{2\delta \beta_{j}}+\frac{\sum_{k=1}^{|C_{-j}|}g_{j,k}t_{k}}{2\delta \beta_{j}}\right\}$$

Furthermore, $U_{j}$ is a continuous and concave function, and its second derivative is negative. Therefore, a Nash equilibrium exists in Stage III.

Next, we prove the uniqueness of the Nash equilibrium in Stage III. Under Assumption 1, we have:

(12) $$-\frac{\partial^{2}U_{j}}{\partial t_{j}^{2}}=2\delta \beta_{j}>\sum_{k=1}^{|C_{-j}|}g_{j,k}=\sum_{k=1}^{|C_{-j}|}\left|g_{j,k}\right|=\left|-\frac{\partial^{2}U_{j}}{\partial t_{j}\partial t_{k}}\right|$$

According to the uniqueness theorem [14,38], the above condition ensures solvability and guarantees that the Nash equilibrium in Stage III is unique.    □

**Theorem** **3.** 
In the second stage, given any transaction fee $f_{ij}^{*}$, the optimal strategy of the electronic device user (i.e., the optimal unit data price) can be determined as follows:

(13) $$p_{ij}^{*}=\frac{\delta \beta_{j}\left(\delta \alpha_{j}+c+f_{ij}-\lambda +b+z\right)+\mu \left(\delta \alpha_{j}+z\right)}{2\delta \beta_{j}+\mu }$$

**Proof.**  According to Theorem 1 and Equation (7), the optimal uploaded data amount $t_{j}^{*}$ can be substituted into Equation (5). Let $z=\sum_{k=1}^{|C_{-j}|}g_{j,k}t_{k}$. Thus, $U_{i}$ can be rewritten as:

(14) $$\begin{array}{cc} U_{i} & =\sum_{j=1}^{\left|C\right|}\left(p_{ij}-f_{ij}\right)t_{j}+\sum_{j=1}^{\left|C\right|}\left(\lambda t_{j}-\mu t_{j}^{2}\right)-\sum_{j=1}^{\left|C\right|}\left(c+b\right)t_{j}\\ \; & =\sum_{j=1}^{\left|C\right|}\left(p_{ij}-f_{ij}\right)\left(\frac{\delta \alpha_{j}-p_{ij}}{2\delta \beta_{j}}+\frac{z}{2\delta \beta_{j}}\right)\\ \; & \;+\sum_{j=1}^{\left|C\right|}\left[\lambda \left(\frac{\delta \alpha_{j}-p_{ij}}{2\delta \beta_{j}}+\frac{z}{2\delta \beta_{j}}\right)-\mu {\left(\frac{\delta \alpha_{j}-p_{ij}}{2\delta \beta_{j}}+\frac{z}{2\delta \beta_{j}}\right)}^{2}\right]\\ \; & \;-\sum_{j=1}^{\left|C\right|}\left(c+b\right)\left(\frac{\delta \alpha_{j}-p_{ij}}{2\delta \beta_{j}}+\frac{z}{2\delta \beta_{j}}\right) \end{array}$$

We then take the first derivative of $U_{i}$ with respect to $p_{ij}$ and set $\frac{\partial U_{i}}{\partial p_{ij}}=0$ to obtain Equation (13).

(15) $$\begin{array}{cc} \frac{\partial U_{i}}{\partial p_{ij}}\; & =\sum_{j=1}^{\left|C\right|}\left(p_{ij}-f_{ij}\right)\left(\frac{\delta \alpha_{j}-p_{ij}}{2\delta \beta_{j}}+\frac{z}{2\delta \beta_{j}}\right)\\ \; & \;+\sum_{j=1}^{\left|C\right|}\left[\lambda \left(\frac{\delta \alpha_{j}-p_{ij}}{2\delta \beta_{j}}+\frac{z}{2\delta \beta_{j}}\right)-\mu {\left(\frac{\delta \alpha_{j}-p_{ij}}{2\delta \beta_{j}}+\frac{z}{2\delta \beta_{j}}\right)}^{2}\right]\\ \; & \;-\sum_{j=1}^{\left|C\right|}\left(c+b\right)\left(\frac{\delta \alpha_{j}-p_{ij}}{2\delta \beta_{j}}+\frac{z}{2\delta \beta_{j}}\right)\\ \; & =\frac{\delta \alpha_{j}-p_{ij}}{2\delta \beta_{j}}+\frac{z}{2\delta \beta_{j}}-\frac{p_{ij}-f_{ij}}{2\delta \beta_{j}}-\frac{\lambda }{2\delta \beta_{j}}\\ \; & \;+2\mu \left(\frac{\delta \alpha_{j}-p_{ij}}{2\delta \beta_{j}}+\frac{z}{2\delta \beta_{j}}\right)\frac{1}{2\delta \beta_{j}}+\frac{c+b}{2\delta \beta_{j}}\\ \; & =\frac{\left(\delta \alpha_{j}-p_{ij}\right)-\left(p_{ij}-f_{ij}\right)-\lambda +c+b}{2\delta \beta_{j}}+\frac{z}{2\delta \beta_{j}}+\frac{\mu (\delta \alpha_{j}-p_{ij})+\mu z}{2\delta^{2}\beta_{j}^{2}} \end{array}$$

Based on the above, the second derivative is

(16) $$\frac{\partial^{2}U_{i}}{\partial p_{ij}^{2}}=-\frac{1}{\delta \beta_{j}}-\frac{\mu }{2\delta^{2}\beta_{j}^{2}}<0$$

□

**Theorem** **4.** 
*In the first stage, the optimal strategy of the intermediator (i.e., the optimal transaction fee) can be expressed as:*

(17) $$f_{ij}^{*}=\frac{\delta \alpha_{j}+z-c+\lambda +s+w-2b}{2}$$

**Proof.**  According to Theorems 1 and 3, Equations (7) and (17), the optimal data purchase volume and the optimal unit data price $f_{ij}^{*}$ can be substituted into Equation (6). Therefore, $U_{pl}$ can be rewritten as:

(18) $$\begin{array}{cc} U_{pl}\; & =\sum_{j=1}^{\left|C\right|}\left(f_{ij}t_{j}-st_{j}\right)+\sum_{j=1}^{\left|C\right|}\left(b-w\right)t_{j}\\ \; & =\sum_{j=1}^{\left|C\right|}\left(f_{ij}-s\right)\left(\frac{\delta \alpha -p_{jt}}{2\delta \beta_{j}}+\frac{z}{2\delta \beta_{j}}\right)+\sum_{j=1}^{\left|C\right|}\left(b-w\right)\left(\frac{\delta \alpha_{j}-p_{jt}}{2\delta \beta_{j}}+\frac{z}{2\delta \beta_{j}}\right)\\ \; & =\sum_{j=1}^{\left|C\right|}\left(f_{ij}-s\right)\left[\frac{\delta \alpha -\frac{\delta \beta \left(\alpha \delta +c+f_{ij}-\lambda +b+z\right)+\mu \left(\alpha_{j}\delta +z\right)}{2\beta_{j}\delta +\mu }}{2\delta \beta_{j}}+\frac{z}{2\delta \beta_{j}}\right]\\ \; & \;+\sum_{j=1}^{\left|C\right|}\left(b-w\right)\left[\frac{\delta \alpha_{j}-\frac{\delta \beta_{j}\left(\alpha_{j}\delta +c+f_{ij}-\lambda +b+z\right)+\mu \left(\alpha_{j}\delta +z\right)}{2\beta_{j}\delta +\mu }}{2\delta \beta_{j}}+\frac{z}{2\delta \beta_{j}}\right] \end{array}$$

Taking the first derivative of $U_{pl}$ with respect to $f_{ij}$ and setting $\frac{\partial U_{pl}}{\partial f_{ij}}=0$, we have:

(19) $$\begin{array}{cc} \frac{\partial U_{pl}}{\partial f_{ij}}\; & =\frac{\delta \alpha_{j}+z}{2\delta \beta_{j}}-\frac{\delta \beta_{j}\left(\alpha_{j}\delta +c+f_{ij}-\lambda +b+z\right)+\mu \left(\alpha_{j}\delta +z\right)}{2\delta \beta_{j}\left(2\beta_{j}\delta +\mu \right)}\\ \; & \;+\frac{\delta \beta_{j}\left(s-f_{ij}-b+w\right)}{2\delta \beta_{j}\left(2\beta_{j}\delta +\mu \right)}\\ \; & =\frac{\left(\delta \alpha_{j}+z\right)\left(2\beta_{j}\delta +\mu \right)-\delta \beta_{j}\left(\alpha_{j}\delta +c+f_{ij}-\lambda +b+z\right)-\mu \left(\alpha_{j}\delta +z\right)+\delta \beta_{j}\left(s-f_{ij}-b+w\right)}{2\delta \beta_{j}\left(2\beta_{j}\delta +\mu \right)}\\ \; & =\frac{\delta \alpha_{j}+z-c+\lambda +s+w-2b-2f_{ij}}{2(2\delta \beta_{j}+\mu )} \end{array}$$

Then, the second derivative is

(20) $$\frac{\partial^{2}U_{pl}}{\partial f_{ij}^{2}}=-\frac{1}{2\delta \beta_{j}+\mu }<0$$

□

Thus, the optimal strategy set $\langle f_{ij}^{*},p_{ij}^{*},t_{j}^{*}\rangle$ can be determined to maximize each participant’s utility. The optimal strategy of each data consumer is affected by its social connections. Data consumers with wider social relationships can obtain greater social benefits, which in turn increase their utility and improve the utility of both the electronic device users and the intermediator.

In the proposed Stackelberg game model, the Nash equilibrium among the intermediator, electronic device users, and data consumers can be obtained by executing Algorithm 1.

**Remark** **1.** 
*The Stackelberg game proposed in this study represents a complete-information incentive mechanism. Parameters in the utility functions (e.g., $\alpha_{j}$, $\beta_{j}$, etc.) are treated as common knowledge. In practical applications, these model parameters can be estimated from historical transaction records or user behavior data. We assume that the intermediator is reliable, allowing electronic device users and data consumers to access this common information through the intermediator. In the present model, social influence is uniformly modeled in a linear form, i.e., it is assumed that interactions among data consumers follow the same functional structure. We acknowledge that in the real world, social effects may exhibit heterogeneity or nonlinearity; however, adopting a linear form in theoretical modeling significantly enhances analytical tractability, enabling closed-form solutions for equilibrium and facilitating subsequent analysis. Similarly, the linear–quadratic structure of the utility functions is chosen to ensure the analytical solvability of the Stackelberg game. It should be emphasized that the linear–quadratic form is not the only possible choice; in specific applications, the utility functions can be adjusted or extended according to practical requirements to improve the model’s expressiveness and applicability.*

### 4.3. A Simple Illustrative Example

We consider a scenario with three data consumers who are socially connected and purchase electrocardiogram (ECG) data from an electronic device user. The initial data purchasing volumes of the three consumers are assumed to be $T=\left\{t_{1},t_{2},t_{3}\right\}=\left\{1.0,\;1.2,\;1.4\right\}.$ Other parameters are set as follows: $\delta =100,\lambda =5,\mu =0.3,b=0.5,c=1,s=5,w=0.3,\alpha_{j}=2,\beta_{j}=0.5.$ Table 2 reports the optimal strategies for each data consumer.
**Algorithm 1:** Optimal Strategy Acquisition for Three-party Stackelberg Game
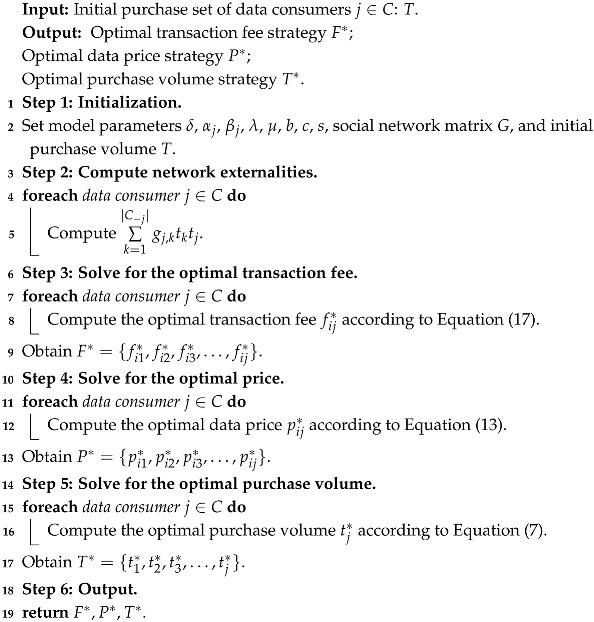


Using Equations (11), (13) and (17), the optimal strategy of data consumer 1 can be computed as:

$$f_{i1}=\frac{100\times 2+1.2\times 0.5-1+5+5+0.3-2\times 0.5}{2}=104.45,$$

$$p_{i1}=\frac{100\times 0.5\times \left(100\times 2+1+104.45-5+0.5+1.2\times 0.5\right)+0.3\times \left(100\times 2+1.2\times 0.5\right)}{2\times 100\times 0.5+0.3}=150.92,$$

$$t_{1}=\frac{100\times 2-150.92+1.2\times 0.5}{2\times 100\times 0.5}=0.4968.$$

The optimal strategy of data consumer 2 is obtained similarly:

$$f_{i2}=\frac{100\times 2+1.0\times 0.5+1.4\times 1.0-1+5+5+0.3-2\times 0.5}{2}=105.1,$$

$$\begin{array}{cc} p_{i2}\; & =\frac{100\times 0.5\times \left(100\times 2+1+105.1-5+0.5+1.0\times 0.5+1.4\times 1.0\right)}{2\times 100\times 0.5+0.3}\\ \; & \;+\frac{0.3\times \left(100\times 2+1.0\times 0.5+1.4\times 1.0\right)}{2\times 100\times 0.5+0.3}=151.9, \end{array}$$

$$t_{2}=\frac{100\times 2-151.9+1.0\times 0.5+1.4\times 1.0}{2\times 100\times 0.5}=0.50.$$

Likewise, the optimal strategy of data consumer 3 is:

$$f_{i3}=\frac{100\times 2+1.2\times 1.0-1+5+5+0.3-2\times 0.5}{2}=104.75,$$

$$p_{i3}=\frac{100\times 0.5\times \left(100\times 2+1+104.75-5+0.5+1.2\times 1.0\right)+0.3\times \left(100\times 2+1.2\times 1.0\right)}{2\times 100\times 0.5+0.3}=151.37,$$

$$t_{3}=\frac{100\times 2-151.37+1.2\times 1.0}{2\times 100\times 0.5}=0.4983.$$

Therefore, the optimal transaction fees, prices, and purchasing volumes are: $F^{*}=\left\{104.45,\;105.1,\;104.75\right\}$, $P^{*}=\left\{150.92,\;151.9,\;151.37\right\}$, $T^{*}=\left\{0.496,\;0.50,\;0.498\right\}$.

## 5. Performance Evaluation and Result Analysis

### 5.1. Impact of Different Social Network Strengths on Performance

We consider four groups of data consumers with different sizes in the social network and set the parameters as follows. We assume that the intrinsic parameters of the data consumers, i.e., $\alpha_{j}$ and $\beta_{j}$, follow normal distributions $N(\mu_{a},0.09)$ and $N(\mu_{b},0.01)$, respectively. In addition, the social relationship $g_{jk}$ between any two data consumers *j* and *k* is modeled as a normal distribution $N(\mu_{g},0.05)$. The default parameter settings are: δ=100, λ=5, μ=0.3, b=0.5, c=1, s=5, w=0.3, $\mu_{a}=2$, $\mu_{b}=0.5$, and $\mu_{g}=0.5$. Note that some of these parameters may vary depending on the evaluation scenario.

A tunable social strength scaling factor γ∈[0,1] is introduced to scale the social network matrix *G*, where γ controls the global intensity of social influence. When γ=0, it represents completely independent decisions without any social network effect; when γ=1, it represents maximum social dependence, meaning that the purchasing behavior of data consumers is strongly influenced by others. In our experimental design, γ is discretized as {0,0.2,0.4,0.6,0.8,1.0} to simulate the gradual increase in social network strength, thereby systematically examining the impact of social interactions on the utilities of electronic device users, the intermediator, and data consumers.

**Figure 2:**
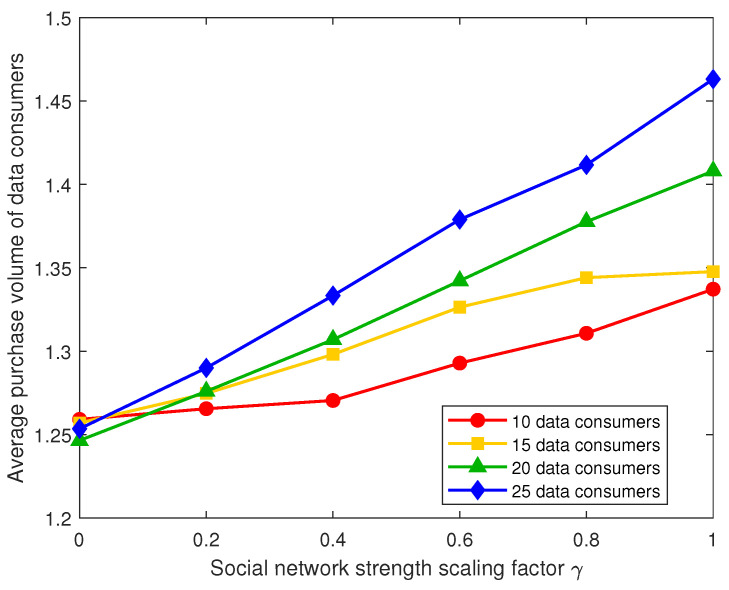
Average purchase volume of data consumers under different social network strengths.

As shown in Figure 2, in the IoT healthcare data-sharing environment, the average purchase volume of data consumers exhibits a significant upward trend with the enhancement of social network strength. Specifically, as the social strength scaling factor increases from 0 to 1, the average purchase volumes increase to varying degrees across all data consumer sizes. This indicates that social network effects play a clear positive incentive role in data-sharing decisions. The underlying reason is that as social connections strengthen, the flow of information and trust among consumers improves, making individual purchase decisions more susceptible to the influence of neighbors or the group, thereby forming a social conformity effect. This positive feedback mechanism allows the proactive purchasing behavior of one consumer to diffuse through the social network, driving an overall increase in purchase levels.

**Figure 3:**
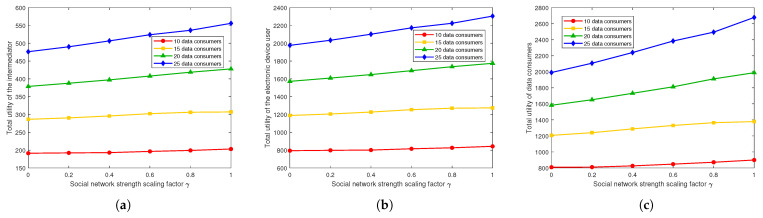
Total utilities of the intermediator, the electronic device user, and data consumers under different social network strengths. (**a**) Total utilities of the intermediator. (**b**) Total utilities of the electronic device user. (**c**) Total utilities of data consumers.

Figure 3a–c illustrate that under different social network strengths, the overall utilities of the intermediator, device users, and data consumers gradually increase with the strengthening of social network relationships. This suggests that reinforcing social network connections can effectively promote the circulation and value realization of IoT healthcare data, thereby enhancing the overall economic efficiency of the system.

In summary, this set of experiments verifies the critical role of social networks in IoT healthcare data sharing. Strong social network effects can effectively promote an increase in consumers’ data purchase volumes and significantly improve their utility levels. Moreover, this effect is more pronounced when the number of data consumers is larger. These findings indicate that to foster sustainable healthcare data sharing and trading, social relationships should be adequately considered in mechanism design to generate positive network externalities, thereby enhancing overall market efficiency and social welfare.

### 5.2. Relationship Among Transaction Fee, Unit Data Price, and Data Purchase Volume

**Figure 4:**
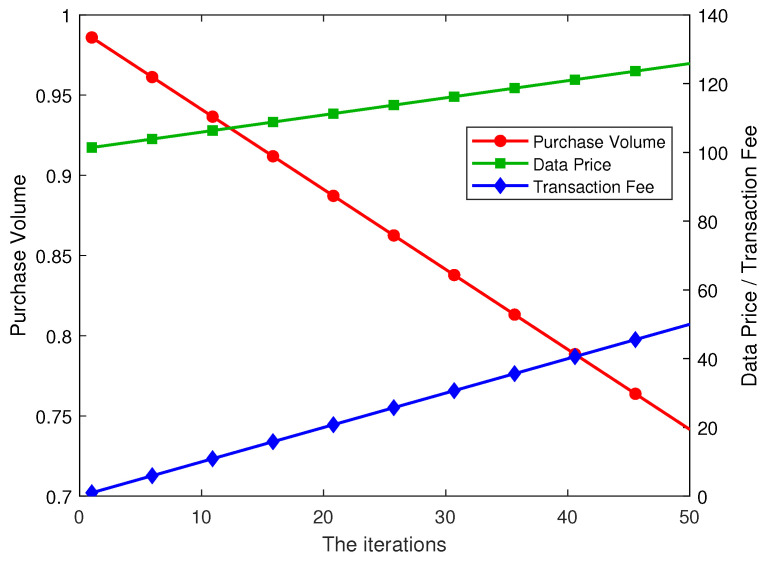
Dynamic relationship among transaction fee, data price, and purchase volume.

Figure 4 illustrates the dynamic relationship among the data purchase volume, unit data price, and transaction fee of data consumers. Overall, the data purchase volume exhibits a decreasing trend as the unit data price increases. Specifically, when the data price continues to rise, data consumers tend to reduce their purchase volume to maintain utility maximization and avoid excessive marginal cost. Conversely, when the unit data price decreases or depreciates, data consumers choose to increase their purchase volume to obtain more data resources at a lower cost, thereby improving their utility level. This adjustment mechanism reflects the optimal response strategy of data consumers when facing price fluctuations.

Meanwhile, changes in the transaction fee indirectly affect the unit data price set by electronic device users. When the intermediator increases the transaction fee, electronic device users tend to raise the unit data price to maintain their utility, thereby transferring part of the cost to data consumers. This price linkage effect further compresses the data consumers’ optimal demand, indicating that the transaction fee not only directly affects the intermediator’s utility but also indirectly influences the equilibrium between data consumers and electronic device users through the price transmission mechanism.

It is noteworthy that the Nash equilibrium formed as the three parties pursue their own utility maximization typically arises under conditions where demand is relatively constrained and both the unit data price and the transaction fee are high. However, the trends illustrated in Figure 4 are primarily based on specific parameter settings, and variations in market scale, data consumer preferences, or price sensitivity may significantly affect the resulting equilibrium outcomes.

From an application perspective, this finding reveals the subtle relationship between price and demand in the IoT-based medical data market. When determining transaction fees, the intermediator needs to balance the indirect impact on device users’ pricing decisions; otherwise, excessive fees may lead to a sharp decline in consumer demand and reduce market activity. Device users should also consider the price sensitivity of data consumers to avoid an abrupt drop in purchase volume. For data consumers, their optimal purchase volume is not only limited by the price level but also influenced by the interaction between transaction fees and device pricing strategies. Therefore, in real market operations, the three parties must strike a balance between maximizing their own utilities and maintaining overall equilibrium stability, while also accounting for potential deviations caused by the model’s limitations. Future research could further incorporate more flexible price formation processes to enhance the practical applicability of the model.

### 5.3. Utility Evaluation of the Intermediator, Electronic Device Users, and Data Consumers

**Figure 5:**
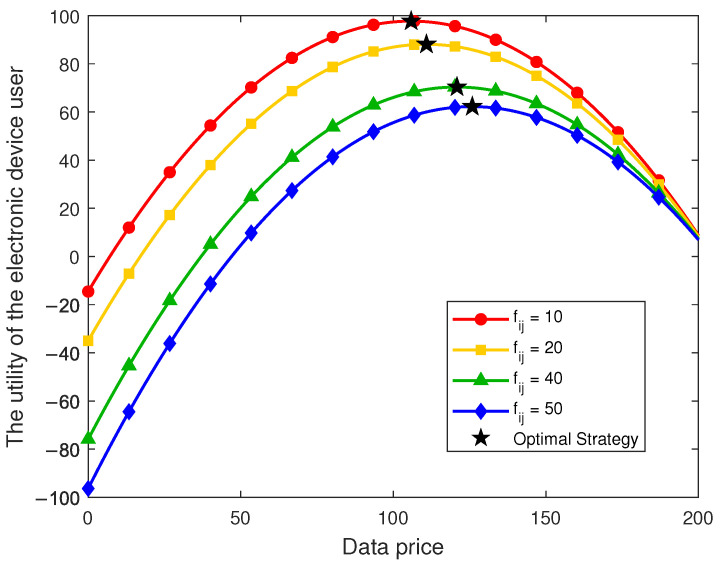
Utility of the electronic device user under different data prices and transaction fees.

Figure 5 illustrates the effect of different unit data prices and transaction fees on the utility of device users. Initially, as the data price increases, the utility of device users decreases and then increases. This trend arises because excessively high data prices discourage data consumers from purchasing data, leading to a reduction in total demand and consequently lowering the utility of device users. Conversely, lower transaction fees reduce trading costs, enabling device users to achieve higher utility levels.

**Figure 6:**
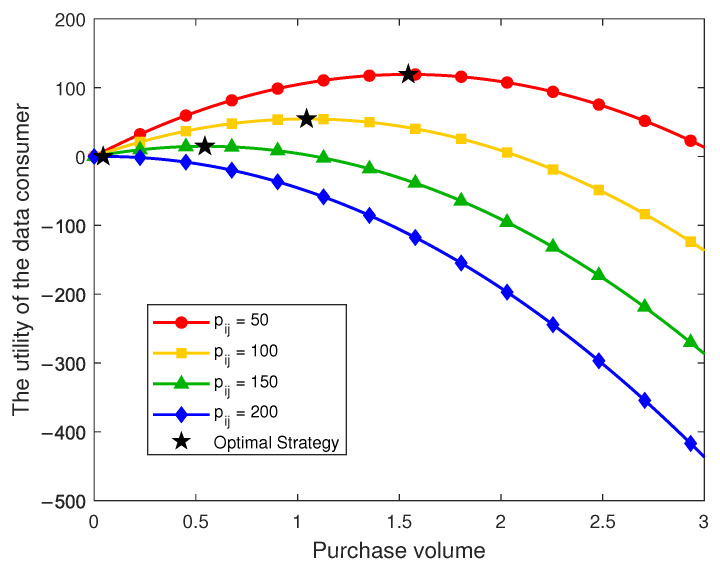
Utility of the data consumer under different data prices and purchase volumes.

Figure 6 systematically presents the dynamic variation of data consumers’ utility with respect to data demand under different data price levels. It can be observed that all curves exhibit an inverted U-shaped trend, indicating the existence of an optimal demand level for each data consumer. When the demand is low, the marginal utility of data consumption exceeds the marginal cost, and thus the overall utility increases with higher demand. However, once the demand surpasses a certain threshold, the marginal cost overtakes the marginal utility, leading to a decline in overall utility. This phenomenon reflects a saturation effect in data demand, implying that higher data consumption does not necessarily yield higher utility; rather, consumers need to choose an appropriate demand level to maximize their utility.

A further comparison across different price levels reveals a significant influence of price on the utility of data consumers. When the data price is set at 50, data consumers achieve the highest overall utility and the largest optimal demand, indicating that lower prices effectively stimulate purchasing behavior. As the price increases to 200, the entire utility curve shifts downward, the maximum utility gradually declines, and the optimal demand shifts leftward. This result suggests that higher data prices not only compress the utility space of consumers but also dampen their purchasing enthusiasm, resulting in a substantially lower optimal demand. These results intuitively illustrate the optimal behavioral choice of data consumers and provide reliable evidence for subsequent pricing and strategy design.

From a macro perspective, the findings verify the theoretical implications of the proposed game model, highlighting that a reasonable data pricing strategy is essential for maintaining market efficiency and ensuring consumer welfare. When the data price is controlled within an appropriate range, it can simultaneously sustain high utility for consumers and promote efficient circulation and utilization of data resources in the long term.

**Figure 7:**
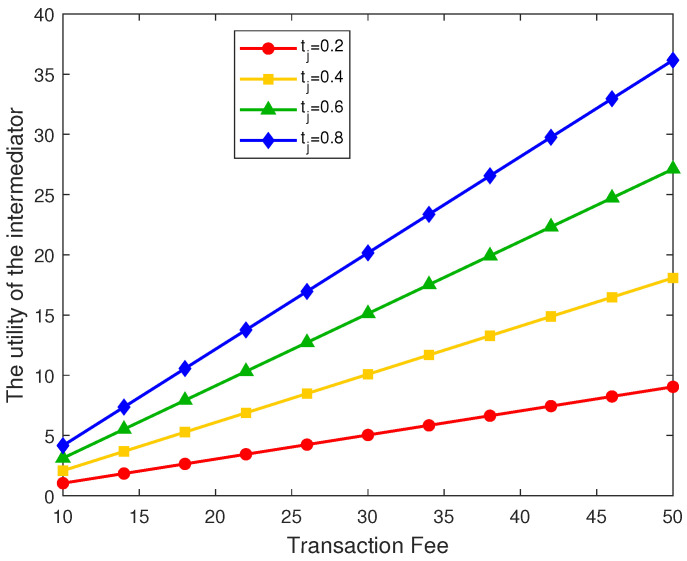
Utility of the intermediator under different transaction fees and purchase volumes.

As shown in Figure 7, when the data consumers’ purchase volume $t_{j}\in \left\{0.2,0.4,0.6,0.8\right\}$ vary, the intermediator’s utility increases almost linearly with the transaction fee $f_{ij}$. The transaction fee refers to the share of revenue the intermediator extracts based on the transaction amount; therefore, the higher the fee, the greater the intermediator’s profit from each transaction. When the purchase volume is high, the slope of the utility curve increases significantly, indicating that strong demand amplifies the intermediator’s marginal revenue gains from raising the transaction fee. However, in real markets, intermediators often face operational management costs, regulatory constraints, and other limiting factors, so the linear growth relationship observed in Figure 7 may be overstated.

Overall, intermediators have greater room to increase their revenue share when data consumers’ purchase volume is high; in contrast, when purchase volume is low, merely raising the transaction fee is insufficient to effectively improve profits. Therefore, enhancing data consumer participation—such as through incentive mechanisms or reinforcing social network effects—remains a crucial pathway for intermediators to optimize long-term utility. It should be noted that the model’s assumption of a static and homogeneous social network effect is a simplification; under more complex and dynamic network structures, the intermediator’s sensitivity to transaction fees may exhibit more diverse patterns.

**Figure 8:**
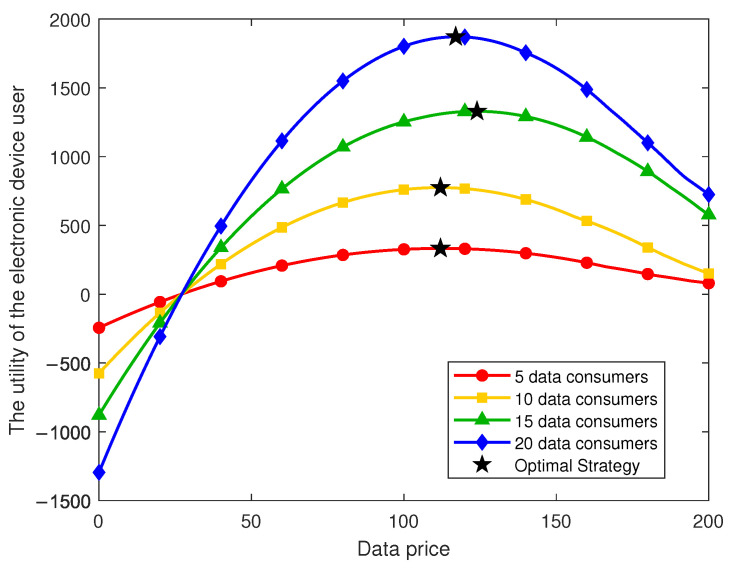
Utility of the electronic device user under different data consumer numbers.

As illustrated in Figure 8, the utility of electronic device users exhibits a typical concave pattern with respect to the unit data price: in the low-price range, an increase in price brings higher returns, but once the price surpasses a certain threshold, the utility begins to decline. This phenomenon indicates that the pricing mechanism exerts a dual effect—moderate price increases enhance the direct revenue of electronic device users, whereas excessively high prices substantially reduce data consumers’ purchase volume, ultimately lowering the users’ overall utility. However, this result also depends on the specific assumptions of the demand function in the model. In this study, price sensitivity is fixed in functional form; thus, the concave structure observed in the figure is partly model-dependent. In real markets, data consumers’ demand elasticity may be more complex, potentially piecewise or heterogeneous, implying that the optimal price may not necessarily exhibit a single peak as shown in the model.

The impact of different numbers of data consumers further supports this observation. As the number of data consumers increases from 5 to 20, the utility curve of electronic device users shifts upward, and the maximum achievable utility rises accordingly. This indicates that market expansion can substantially strengthen the incentives for electronic device users. Nevertheless, the model assumes relatively homogeneous preferences and behaviors among all data consumers, whereas, in real IoT healthcare ecosystems, consumer heterogeneity is substantial. Such heterogeneity may cause the magnitude of utility gains to scale nonlinearly and may also shift the optimal pricing point. Therefore, the scale effects presented in Figure 8 may overestimate the utility improvements associated with an increased number of data consumers in actual markets.

Overall, the results in Figure 8 highlight the critical role of appropriate pricing and a larger participant base in IoT medical data sharing. A properly chosen data price can effectively incentivize electronic device users to contribute data, while avoiding the reduction in market liquidity caused by excessive pricing. Additionally, attracting more data consumers—such as research institutions or third-party service providers—can significantly enhance market demand and improve overall utility. Therefore, multi-agent participation and well-designed pricing strategies jointly form the core mechanism that drives the realization of data value, providing important guidance for future pricing and incentive design in electronic medical data sharing.

### 5.4. Parameter Sensitivity Analysis

**Figure 9:**
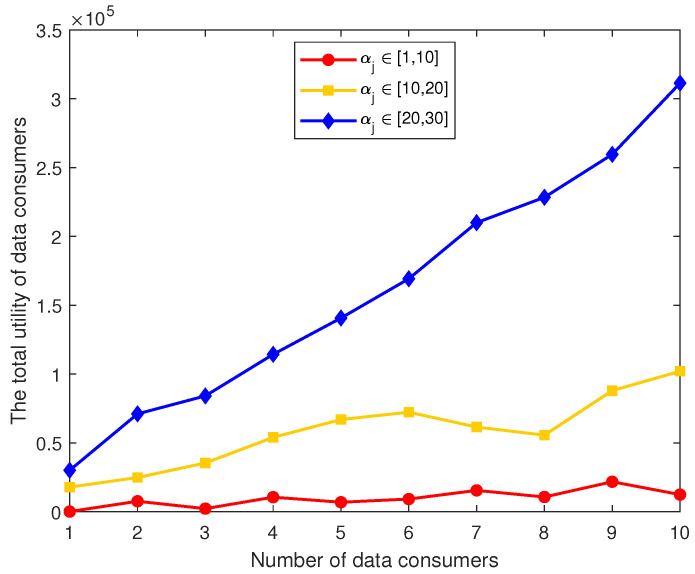
Total utility of data consumers under different $\alpha_{j}$ intervals as the number of consumers increases.

The parameter $\alpha_{j}$ has a significant impact on the utility of data consumers. The variation trend of the optimal utility for data consumers with different $\alpha_{j}$ values is illustrated in Figure 9. Specifically, Figure 9 depicts the total utility of the data consumer group as the number of consumers increases across different $\alpha_{j}$ intervals.

First, as the number of consumers increases, the aggregate utility exhibits an upward trend, indicating that higher participation can generate greater socialized data value. Second, notable differences in utility levels are observed across the $\alpha_{j}$ intervals. Data consumers with $\alpha_{j}\in \left[20,30\right]$ achieve substantially higher utility compared to the other intervals, suggesting that greater data utilization capability accelerates utility growth. Conversely, for $\alpha_{j}\in \left[1,10\right]$, the utility remains relatively low and increases gradually, implying that limited data utilization capability constrains consumers’ incentives to purchase data. The case of $\alpha_{j}\in \left[10,20\right]$ lies between these extremes, demonstrating moderate utility growth.

However, interpreting the model’s performance solely based on graphical trends remains limited. First, the result depends on the linear–quadratic utility specification, which causes the utility growth of high-capability groups to exhibit an idealized linear enhancement. In real-world settings, capability improvement may face diminishing marginal returns or regulatory constraints, meaning that actual growth may be far less smooth. Second, the model assumes that data consumers do not face resource competition or budget constraints, which may lead to an overestimation of the utility gains for high-capability groups. Future work may incorporate more realistic network structures, capability distributions, and cost constraints to more comprehensively evaluate the impact of this parameter in real data-sharing markets.

Overall, these results confirm the critical role of the parameter $\alpha_{j}$ in shaping data consumer utility, and its interval directly influences the utility performance of the consumer group at different scales. These findings provide strong support for further investigation into the impact of heterogeneous characteristics of data consumers on the data-sharing market.

## 6. Conclusions

This study focuses on incentive mechanisms for IoT-based medical data sharing and develops an analytical framework based on a three-party Stackelberg game, systematically characterizing the multi-level interactions among the intermediator, IoT device users (data providers), and data consumers. By incorporating social network externalities, the model captures the propagation and influence of data demand across different groups, thereby more realistically reflecting the social connections and decision interdependencies present in practical medical data-sharing scenarios. Theoretical analysis and numerical simulations demonstrate that the proposed mechanism not only effectively enhances data consumers’ purchasing willingness and data acquisition levels but also maintains a dynamic balance of utility among the intermediator, device users, and data consumers. The present study further extends the game-theoretic modeling framework for IoT-based medical data sharing and provides valuable practical insights for the design and implementation of incentive mechanisms in IoT healthcare systems.

From a practical deployment perspective, the incentive mechanism proposed in this study has certain applicability in medical data sharing platforms. First, the three-party Stackelberg framework naturally maps to the hierarchical roles in real-world medical data flows, including the intermediator setting transaction fees and revenue-sharing policies, data providers making pricing decisions, and data consumers responding to demand under social influence. Second, the parameters and decision variables used in this study can be estimated from data transaction records or upload behaviors from wearable devices, providing an operational basis for mechanism deployment. Moreover, the model results offer insights for privacy regulations; for instance, the intermediator can enhance user trust through more transparent transaction fees and improve data providers’ participation stability through dynamic incentives. At the same time, regulators can leverage the structured game-theoretic framework proposed here to analyze potential incentive imbalances and market manipulation risks in data flows, thereby formulating more reasonable data sharing standards and policies.

Nevertheless, this study has certain limitations. For example, it does not incorporate multi-dimensional heterogeneity factors such as privacy sensitivity perturbations and user heterogeneity. The model is still based on a relatively static mechanism design and does not cover dynamic incentives and evolutionary features under long-term interactions. In addition, regarding incentive mechanisms for IoT healthcare data sharing, few studies in the existing literature provide publicly available real transaction data or case studies. Due to data privacy and accessibility constraints, this study is also unable to obtain real transaction information, and therefore mainly relies on theoretical modeling and simulation analysis for validation. This approach is common in this research field, and even in related popular areas such as mobile crowdsourcing, publicly available real case analyses are rare.

Future research could further expand in the following directions: constructing a unified experimental platform with higher generalizability to enable systematic comparison with existing incentive methods; incorporating robustness against privacy perturbations, user heterogeneity, and intermediator strategy manipulations; and exploring more complex market structures, such as multi-alliance and multi-intermediator competition, to provide more comprehensive theoretical support and policy guidance for efficient, secure, and sustainable sharing of medical data.

## Figures and Tables

**Table 1:** Explanation of main symbols.

Symbol	Description
C,|C|	Set of data consumers and the number of data consumers.
$C_{-j},\left|C_{-j}\right|$	Set of data consumers excluding consumer *j* and its size.
δ	Adjustable coefficient for data consumer utility.
$\alpha_{j},\beta_{j}$	Data utility coefficients of data consumer *j*.
λ,μ	Social benefit coefficients obtained by the electronic device user.
*b*	Unit data hosting fee that the electronic device user pays to the intermediator.
*c*	The comprehensive cost incurred by an electronic device user to collect and share a single unit of health data, including time, effort, and energy consumption for sensor data acquisition.
*s*	Transmission cost of the intermediator.
*w*	Unit data storage cost of the intermediator.
$f_{ij}$	Transaction fee charged by the intermediator, i.e., a fee deducted from each data transaction to compensate for data management and operational costs.
$p_{ij}$	Unit data price set by the electronic device user.
$t_{j}$	Data purchase volume of data consumer *j*.
$U_{pl}$	Utility function of the intermediator.
$U_{i}$	Utility function of the electronic device user.
$U_{j}$	Utility function of data consumers.

**Table 2:** Optimal strategies for each data consumer.

Data Consumer	Social Network Effects	Optimal Strategy
Consumer 1	$g_{12}=0.5,\;g_{13}=0$	$f_{i1}=104.45,\;p_{i1}=150.92,\;t_{1}=0.496$
Consumer 2	$g_{21}=0.5,\;g_{23}=1$	$f_{i2}=105.1,\;p_{i2}=151.9,\;t_{2}=0.50$
Consumer 3	$g_{31}=0,\;g_{32}=1$	$f_{i3}=104.75,\;p_{i3}=151.37,\;t_{3}=0.498$

## Data Availability

Data are contained within the article.
